# Inhibition of Neurotoxic Secretory Phospholipases A_2_ Enzymatic, Edematogenic, and Myotoxic Activities by Harpalycin 2, an Isoflavone Isolated from *Harpalyce brasiliana* Benth

**DOI:** 10.1155/2012/987517

**Published:** 2012-07-31

**Authors:** Rafael M. Ximenes, Marcelo M. Rabello, Renata M. Araújo, Edilberto R. Silveira, Fábio H. R. Fagundes, Eduardo B. S. Diz-Filho, Simone C. Buzzo, Veronica C. G. Soares, Daniela de O. Toyama, Henrique H. Gaeta, Marcelo Z. Hernandes, Helena S. A. Monteiro, Marcos H. Toyama

**Affiliations:** ^1^Departamento de Fisiologia e Farmacologia, Universidade Federal do Ceará, Rua Coronel Nunes de Melo 1315, 60430-270 Fortaleza, CE, Brazil; ^2^Departamento de Ciências Farmacêuticas, Universidade Federal de Pernambuco, 50740-520 Recife, PE, Brazil; ^3^Departamento de Química, Universidade Federal do Rio Grande do Norte, 50078-970 Natal, RN, Brazil; ^4^Centro Nordestino de Aplicação e Uso da Ressonância Magnética Nuclear (CENAUREN), Universidade Federal do Ceará, 60455-760 Fortaleza, CE, Brazil; ^5^Departamento de Bioquímica, Instituto de Biomedicina, Universidade de Campinas, 13082-862 Campinas, SP, Brazil; ^6^Unidade de São Vicente, Campus do Litoral Paulista, Universidade Estadual Paulista Júlio Mesquita Filho, 11330-900 São Vicente, SP, Brazil; ^7^Centro de Ciências Biológicas e da Saúde, Universidade Presbiteriana Mackenzie, 01302-970 São Paulo, SP, Brazil

## Abstract

Secretory phospholipases A_2_ (sPLA_2_) exert proinflammatory actions through lipid mediators. These enzymes have been found to be elevated in many inflammatory disorders such as rheumatoid arthritis, sepsis, and atherosclerosis. The aim of this study was to evaluate the effect of harpalycin 2 (Har2), an isoflavone isolated from *Harpalyce brasiliana* Benth., in the enzymatic, edematogenic, and myotoxic activities of sPLA_2_ from *Bothrops pirajai, Crotalus durissus terrificus, Apis mellifera,* and *Naja naja* venoms. Har2 inhibits all sPLA_2_ tested. PrTX-III (*B. pirajai* venom) was inhibited at about 58.7%, Cdt F15 (*C. d. terrificus* venom) at 78.8%, Apis (from bee venom) at 87.7%, and Naja (*N. naja* venom) at 88.1%. Edema induced by exogenous sPLA_2_ administration performed in mice paws showed significant inhibition by Har2 at the initial step. In addition, Har2 also inhibited the myotoxic activity of these sPLA_2_s. In order to understand how Har2 interacts with these enzymes, docking calculations were made, indicating that the residues His48 and Asp49 in the active site of these enzymes interacted powerfully with Har2 through hydrogen bonds. These data pointed to a possible anti-inflammatory activity of Har2 through sPLA_2_ inhibition.

## 1. Introduction

The flavonoids are a group of plant secondary metabolites widely distributed in nature. They are divided in two main classes: the flavonoids and the isoflavonoids. Many pharmacological activities have been described for both classes such as anti-inflammatory and antioxidant, antiallergic, antiviral, and anticancer [1, 2]. Many works have shown the phospholipase A_2_ (PLA2) inhibitory effects of flavonoids such as rutin [3], morin [4], and quercetin [5], however, only few works have been shown the antiphospholipasic A_2_ activity of isoflavonoids [6].

 The PLA_2_ s are a class of enzymes (EC 3.1.1.4) that catalyzes the hydrolysis of the sn-2 ester bond of phospholipids to produce lysophospholipids and free fatty acids, which are substrates for the synthesis of proinflammatory eicosanoids and platelet aggregating factor (PAF) [7, 8], in addition to reactive oxygen species produced during the synthesis of eicosanoids (which play a role as a positive feedback of the enzymatic active of the PLA_2_) [9]. They are divided in two major groups: cytosolic phospholipase A_2_ (cPLA_2_) and secretory phospholipase A_2_ (sPLA_2_). The last are divided in ten groups as discussed elsewhere [10]. In many inflammatory diseases, as in sepsis, atherosclerosis, and rheumatoid arthritis, group IIA sPLA_2_ are found in the inflammatory areas and play a role not fully understood up to date [11, 12].

 The sPLA_2_ present in animal venoms share structural features with mammalian (including human) group IIA sPLA_2_, mainly in the active site, being for that reason useful tools for the study of sPLA_2_ inhibitors [7–12]. The exogenous administration of these enzymes to experimental animals provokes an inflammatory response similar to that observed with administration of endogenous sPLA_2_, besides more specific responses as myonecrosis [5, 13].

Current anti-inflammatory therapies include nonsteroidal anti-inflammatory drugs that inhibit either LOX or COX-1/2 enzymes and have serious side effects such as gastrointestinal ulceration, bleeding, and cardiovascular complications. In addition to these problems, COX-1/2 and LOX inhibitors cannot regulate the production of the PAF, which continues causing inflammation [7, 8]. Effective inhibitors of sPLA_2_ could be capable of depleting the downstream proinflammatory metabolites of arachidonic acid as well as PAF, without the adverse effects of the current corticosteroids therapy since these enzymes are secreted only in pathological conditions [7–10].

*Harpalyce brasiliana* Benth. (Fabaceae) is a Brazilian folk medicine, popularly known as “raiz-de-cobra” (Port. Lit. *snakeroot*). Its roots have been used in the Northeast of Brazil to treat snakebite [14], while its leaves are claimed to be anti-inflammatory (personal unpublished data). In this paper, the effect of harpalycin 2 in structure and enzymatic, edematogenic, and myotoxic activities of four sPLA_2_ isolated from animal venoms was evaluated. In addition, the analysis of the interaction between harpalycin 2 and the active site of the tested sPLA_2_ was performed by docking calculations.

## 2. Material and Methods

### 2.1. Material

Secretory PLA_2_ from *Bothrops pirajai* (PrTx-III) and *Crotalus durissus terrificus* (Cdt F15) were purified as described by Toyama et al. [15, 16], respectively. sPLA_2_ from *Apis mellifera *venom was purchased from BIOMOL International. Bovine pancreas and *Naja naja* venom sPLA_2_ were purchased from Sigma-Aldrich. The COX-1, COX-2, LOX 15hrc, and LOX 15syP1 came from Cayman Chemical. Other salts, reagents, solvents were ultrapure grade, HPLC grade, or sequencing grade purchase from the BIORAD, Sigma-Aldrich (Supelco) and Pharmacia.

### 2.2. Plant Material

Leaves of *Harpalyce brasiliana *Benth. were collected at the Chapada do Araripe, Barbalha (Ceará, Brazil) by Prof. E. R. Silveira. Botanical authentication was made by Prof. E. P. Nunes of the Department of Biology, Federal University of Ceará. Voucher specimen (number: 32525) has been deposited at the Prisco Bezerra Herbarium (EAC), Department de Biology, Federal University of Ceará, Fortaleza (Ceará, Brazil).

### 2.3. General Procedures

The mass spectra were obtained on a Hewlett-Packard 5971 mass spectrometer by electron impact ionization (70 eV). ^1^H and ^13^C NMR spectra were recorded on a Bruker Avance DRX-500 (500 MHz for 1H and 125 MHz for 13C); chemical shifts were expressedin scale and were referenced to residual DMSO (2.5 and 39.5 ppm). Silica Gel 60 (Merck, 70–230 mesh) was used for analytical TLC. Column chromatographies were performed over silica gel (Merck, 60 F254 230–400 mesh).

### 2.4. Extraction and Isolation of Harpalycin 2

Leaves of *Harpalyce brasiliana *were pulverized and extracted with EtOH at room temperature. The solvent was removed under reduced pressure which produced a dark viscous extract (HBFE). Liquid-liquid partition of a water suspension of HBFE (110 g) using petrol ether, CHCl_3_, EtOAc, and n-BuOH yielded five fractions after solvent evaporation: HBFEEp (24.5 g), HBFEC (22.4 g), HBFEA (6.8 g), HBFEB (30.4 g), and HBFEAq (21.2 g).

Flash chromatography of HBFEC (12.0 g) using n-hexane and EtOAc as binary mixtures of increasing polarity afforded 30 fractions, which were pooled in 9 fractions after thin layer chromatography (TLC) analysis. HBFEC (10–12) presented a yellow precipitate, yielding 120.0 mg after recrystallization. NMR and Mass-spectrometric analysis showed the structure of the isoflavone harpalycin 2 (Har2). The fractions HBFEC (8-9) and HBFEC (13–17) were purified, using the same method, yielding more 200.0 mg of Har2.

### 2.5. Inhibition of sPLA_2_ Activity

sPLA_2_ activity was measured following the protocols described by Hernandez-Oliveira et al. [17] and modified by Toyama et al. [18] for 96-well plate. The standard assay mixture contained 200 mL of buffer (10 mM Tris-HCl, 10 mM CaCl_2_, 100 mM, and NaCl, pH 7.8), 20 *μ*L of substrate (4-nitro-3-octanoyloxy-benzoic acid (4N3OBA) 1 mg/mL, manufactured by BIOMOL, USA), 20 *μ*L of water, and 20 *μ*L of sPLA_2_ solution (1 mg/mL). Enzymatic activity was calculated based on the increase in absorbance at 425 nm after 20 min, at 37°C, as a direct result of the cleavage of the synthetic substrate. All assays were done using *n* = 12 and absorbance was measured using a SpectraMax 340 multiwell plate reader (Molecular Devices, Sunnyvale, CA). Evaluation of Har2 effect on sPLA_2_ enzymatic activity was performed after incubation of *Bothrops pirajai* (PrTx-III), *Crotalus durissis terrificus* (Cdt F15), *Apis mellifera* (purified, without the mellitin component), and *Naja naja* sPLA_2_ with Har2 at equal mass (1 : 1; w : w) for a period of 30 minutes. The final concentration of the inhibitor in the reaction mixture was the same of the substrate due to the kinetic behavior of the sPLA_2_.

### 2.6. Animals

Male Swiss mice (20–25 g) obtained from the Animal Facilities of Federal University of Ceará were used in this study. The animals were maintained under standard conditions (22 ± 2°C; 12 h light/dark cycle) with food and water *ad libitum*. All experiments with animals were guided in accordance with Brazilian laws for Care and Use of Laboratory Animals and all the study protocols were approved by Committee of Ethics from Federal University of Ceará (Fortaleza, Brazil) protocol number 68/08.

### 2.7. Neutralization of the Edema Inducing Activity

Neutralization of sPLA_2_-induced paw edema by Har2 was performed according to Iglesias et al. [4], using male Swiss mice (20–25 g, *n* = 6). The edema was induced by a single subplantar injection of 25 *μ*L of sPLA_2_ (25 *μ*g/paw). Paw volume was measured immediately before the injection of the samples and at selected time intervals thereafter (30, 60, 120, 240, and 480 minutes) using a plethysmometer (Ugo Basile, Italy). All samples were dissolved in sterile PBS. Results were expressed as the increase in paw volume (*μ*L) and calculated by subtracting the basal volume. Evaluation of Har2 effect on sPLA_2_ edema-inducing activity was carried out after incubation of *Bothrops pirajai* (PrTx-III), *Crotalus durissis terrificus* (Cdt F15), *Apis mellifera* (purified, without the mellitin component), and *Naja naja* sPLA_2_ with Har2 at equal mass (1 : 1; w : w) for 30 minutes at 37°C. The negative controls were performed by administration of Har2 (25 *μ*g/paw). These values were subtracted from the volume of the paws treated with sPLA_2_ incubated with Har2 for clarity reasons.

### 2.8. Neutralization of Myotoxic Activity

Plasma creatine kinase (CK) activity was measured using a CK-UV kinetic kit (Sigma Chemical Co.). Native sPLA_2_ (1 mg/mL) or those previously incubated with Har2, as described above, were injected intramuscularly (25 *μ*L) in the gastrocnemius of male Swiss mice (20–25 g, *n* = 6). The control group was injected with sterile PBS, and the negative control with Har2. After 3 hours, a blood sample was collected from the tails using heparinized capillary tubes and centrifuged for plasma separation. CK activity was determined in triplicate using 4 *μ*L of plasma according to the manufacturer's instructions, and its activity was expressed in U/L.

### 2.9. Characterization of IC_50_ of Har2 against Several sPLA_2_ and Inflammatory Enzymes

The inhibitory capability of Har2 against COX-1/2, LOX 15hrc, and 15syP1, bovine pancreas, and human group V sPLA_2_ was investigated according to the manufacturer's instructions (Cayman Chemical). All assays were carried out using *n* = 12 and the data measured using a SpectraMax 340 multiwell plate reader (Molecular Devices, Sunnyvale, CA). Har2 was added in different concentrations and IC_50_ values were calculated using GraphPad Prism 5.0.

### 2.10. Circular Dichroism Spectroscopy

Native sPLA_2_, and Har2-treated sPLA2 were dissolved in 10 mM sodium phosphate buffer (pH 7.4) and final protein concentrations were adjusted to 8.7 mM. After centrifugation at 4000 g for 5 min, samples of 20 *μ*L were injected into molecular exclusion column TSK G4000SWXL (0.7 × 300 mm) coupled in the LC-2000Plus Series HPLC Systems (Jasco, USA), which have been previously equilibrated with same buffer used for the preparation of the samples sPLA_2_, Har2-treated sPLA_2_. In this case, the chromatographic run of each samples was simultaneously monitored using a CD-2095 Circular Dichroism HPLC detector (Jasco, USA), FP-2020 Fluorescence detector, and UV-2075 190 to 600 nm detector. Circular dichroism spectra were obtained by adjust the wavelength range 220–260 nm and 260–320 nm to measure the presence of random coil and tertiary protein folding, respectively. Data collection was performed with a bandwidth of 1 nm, response time of 1 s at room temperature with 100 nm/min scanning speed. The fluorescence detection was adjusted specifically for monitoring the fluorescence emission of tryptophan, which was measured between 300 and 450 nm after excitation at 280 nm.

### 2.11. Molecular Modeling (Docking)

The structural optimization of the harpalycin 2 ligand was initially achieved using the AM1 method [19] implemented in the BioMedCache program (BioMedCache, 1989) with default values for the convergence criteria. Docking calculations were performed with the GOLD 4.0 program [20] in order to obtain the relative *in silico* affinities of the Har2 ligand with respect to the sPLA_2_ targets. The sPLA_2_ structures were taken from the RCSB Protein Data Bank (http://www.pdb.org/), under the PDB ID: 1GMZ, 2QOG, 1PSH, and 1POC, respectively, for PrTX-III, Cdt F15, Naja, and Apis.

The docking calculations were performed taking advantage of the flexibility of the Har2 ligand, by activating its rotational degrees of freedom. The active site was defined as all atoms within a radius of 10.0 Å from the residue 48 (His or Asp), which is an important residue according to the literature [21, 22].

### 2.12. Statistical Analysis

Results were expressed as the mean ± SEM of replicated experiments. The significance of differences between means was assessed by an analysis of variance, followed by a Dunnett's test where several experimental groups were compared with the control group. The confidence limit for significance was set at *P* < 0.05.

## 3. Results and Discussion

**Figure 1:**
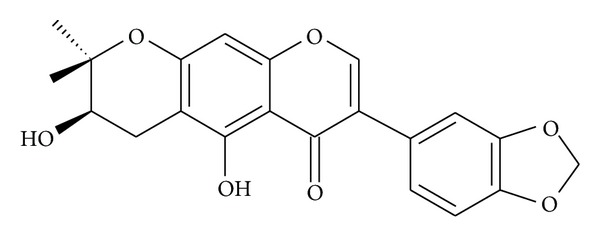
The structure of harpalycin 2.

Natural products from plants are of potential interest for the treatment of a number of inflammatory diseases. They serve as template molecules for the development of new drugs and prototypes [12]. Flavonoids are polyphenolic compounds widely distributed in plants. Due to their various effects on immune and inflammatory systems, these compounds are currently of great pharmacological interest [3]. There are several reports demonstrating that flavonoids are able to inhibit PLA_2_ activity, arachidonic acid release, and the formation of arachidonic acid metabolites [3, 4, 13]. In particular, isoflavones have also been reported to show anti-inflammatory activities, including inhibition of phospholipases A_2_ and COX-1/2 [17, 18]. Harpalycin 2 (Har2) was isolated as a white amorphous solid with m.p. 232.6–234.4°C. Its molecular formula of C_21_H_18_O_7_ was established by the molecular ion at m/z 382 Daltons in the MS spectrum. Structure elucidation was performed by spectroscopic means, including 1D and 2D NMR, and comparison with the data from literature [14]. The structure of Har2 is shown in Figure 1.

**Figure 2:**
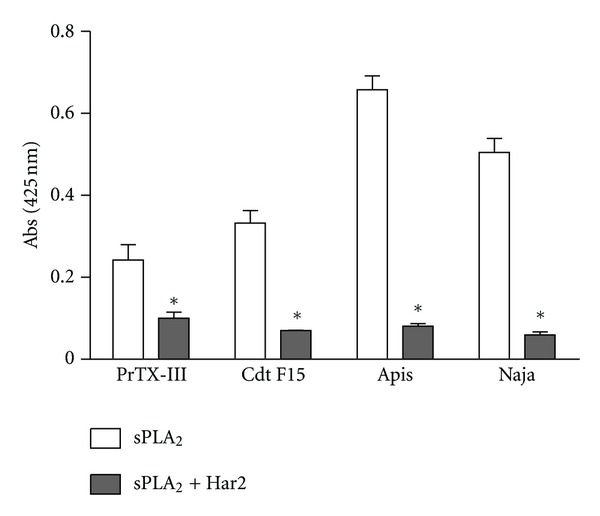
Effect of harpalycin 2 (Har2) on enzymatic activity of sPLA_2_ from *Bothrops pirajai* (PrTX-III), *Crotalus durissus terrificus* (Cdt F15), *Apis mellifera* (Apis), and *Naja naja* (Naja) venom expressed as initial velocity of reaction after 20 min (Vo). (a) Native sPLA_2_ enzymatic activity and (b) harpalycin 2 treated sPLA_2_ enzymatic activity. Data expressed as mean ± S.E.M. and analyzed by ANOVA followed by Dunnett's test, with *P set at 0.05.

Group IIA secretory phospholipases A_2_ may be catalytically active or inactive depending on the amino acid residue 49. In catalytically active isoforms, this residue is occupied by an aspartic acid [24]. Cotrim et al. [5] showed through docking calculations that the chemical treatment of crotoxin B (Cdt F15) with quercetin lead to an inhibition of enzymatic activity due to the fact that quercetin binds in the vicinity of His48 and Asp49 residues. Herein, Har2 inhibits all sPLA_2_ tested when the treatment was made before the substrate addiction, with percentages of inhibition at about 58.7% for PrTX-III, at 78.8% for Cdt F15, at 87.7% for Apis, and at 88.1% for Naja secretory phospholipase A_2_ (Figure 2). These percentages of inhibition were greater than p-bromophenacyl bromide (~40%), a well-known sPLA_2_ inhibitor [5].

**Figure 3:**
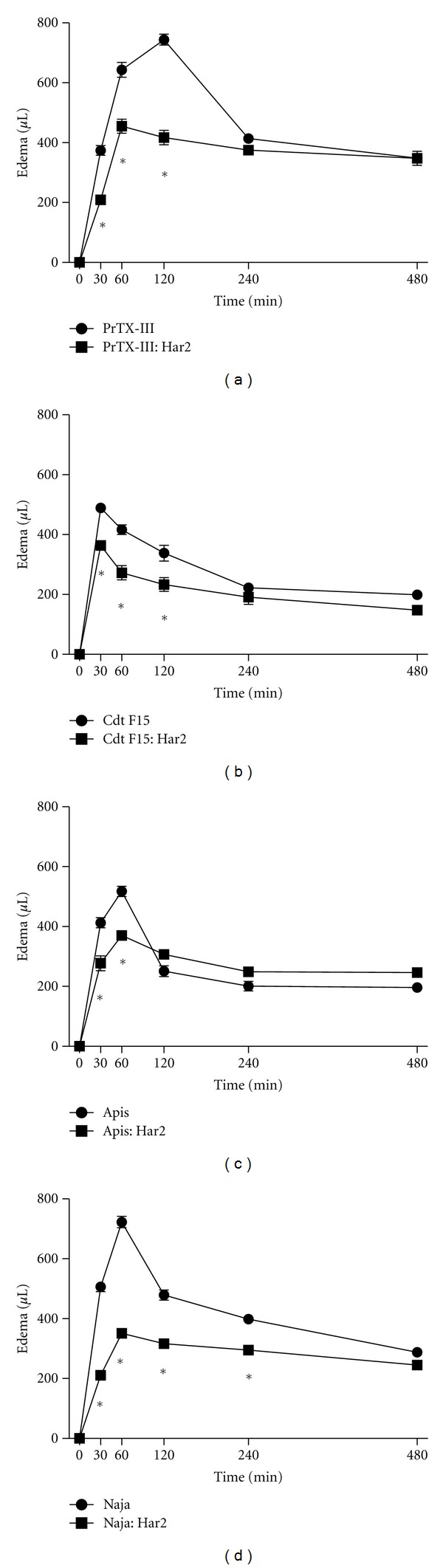
Effect of harpalycin 2 (Har2) on edema formation after a single subplantar injection of sPLA_2_ (25 *μ*g/paw) from (a) *Bothrops pirajai* (PrTX-III), (b) *Crotalus durissus terrificus* (Cdt F15), (c) *Apis mellifera* (Apis), and (d) *Naja naja* (Naja) venom expressed as the increase in paw volume (*μ*L). Native sPLA_2_ edematogenic activity is represented by circles whereas harpalycin 2 treated sPLA_2_ edematogenic activity is showed as squares. Data expressed as mean ± S.E.M. and analyzed by ANOVA followed by Dunnett's test, with *P set at 0.05.

Neutralization of edema induced by exogenous sPLA_2_ administration performed in mice paws by harpalycin 2 showed significant inhibition of the edema initial step induced by PrTX-III, Cdt F15, Apis, and Naja (Figure 3). This first step is correlated with histamine/serotonin involvement [24]. The edema-inducing effect of sPLA_2_ s could be attributed to their ability to hydrolyse phospholipids; however Lys49 phospholipase A_2_-homologues can also induce edema in the absence of PLA_2_ activity, which implies a different mechanism of action for this pharmacological effect [24]. In this case, all sPLA_2_ tested were catalytic active and the partial inhibition of edema formation may be correlated to the inhibition of phospholipids catalysis by the isoflavone.

**Figure 4:**
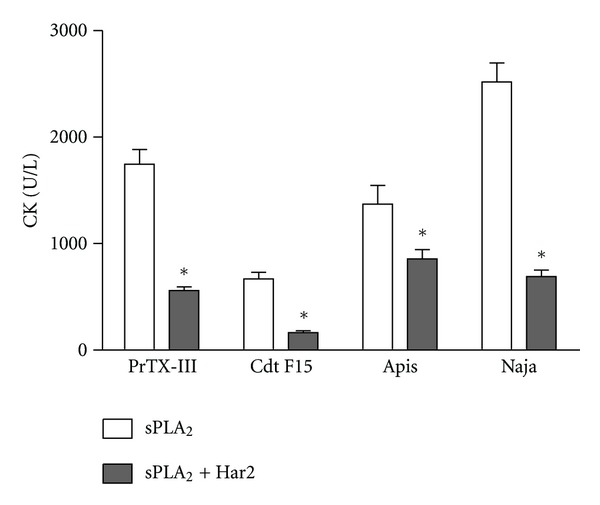
Inhibition of myotoxic activity of sPLA_2_ (25 *μ*g/mice) from *Bothrops pirajai* (PrTX-III), *Crotalus durissus terrificus* (Cdt F15), *Apis mellifera* (Apis) and *Naja naja* (Naja) venom expressed as creatine kinase release on plasma. (a) Native sPLA_2_ myotoxicity and (b) harpalycin 2 (Har2) treated sPLA_2_ myotoxicity. Data expressed as mean ± S.E.M. and analyzed by ANOVA followed by Dunnett's test, with *P set at 0.05.

Harpalycin 2 was able to inhibit the myotoxic activity of the venom secretory phospholipases A_2_ tested in this study (Figure 4), which is a pharmacological activity shared by several types of snake venom sPLA_2_ [25]. This model, when studied using Asp49 sPLA_2_, is interesting for evaluation of the interfacial activation of the sPLA_2_s, since myotoxicity is directed linked with enzymatic activity in the catalytic sPLA_2_ isoforms [7]. Recently, involvement of potassium, ATP, calcium, and purinergic receptors in the myotoxic activity of snake venom sPLA_2_ was described by Cintra-Francischinelli et al. [23]. The authors emphasized the role of the purinergic receptor P2X and its inhibitors in the extent of muscle tissue damage, giving an additional explanation to the finding that the antitrypanosomal drug suramin provides protection from the toxic effect of the Lys49 myotoxins of the *Bothrops jararacussu* and *Bothrops asper* venoms. Suramin also binds to P2X channels, and this property could, at least in part, account for its myotoxic inhibitory activity. Several flavonoids and isoflavones antagonize purinergic receptors [26] which could explain in part the inhibitory effect of these compounds in the myotoxic activity of snake venom sPLA_2_.

In order to elucidate the anti-inflammatory activity of harpalycin 2, we further examined the ability of Har2 to inhibit the enzymatic activity of COX-1/2 and LOX 15hrc and 15syP1 as well as bovine pancreas PLA_2_ and human group V PLA_2_. Har2 showed lower IC_50_ values for both bovine and human sPLA_2_ than for COX-1/2 and LOX enzymes, as shown in Table 1. These results showed that harpalycin 2 has probably more affinity for sPLA_2_ than for other proinflammatory enzymes.

Aiming to understand how harpalycin 2 interacts with these sPLA_2_s, CD spectroscopy, fluorescence analysis, and docking calculations were performed. Fluorescence profile analysis of Har2, sPLA_2_s, and Har2:sPLA_2_s showed significant changes in the spectral fluorescence profile among Har2 and the sPLA_2_ treated with Har2. CD analysis of native and Har2-treated sPLA_2_ revealed that the treatment induced discrete unfolding of sPLA_2_, which did not modify the tridimensional structure of the proteins (See supplementary material, available at doi: 10.1155/2012/987517).

**Figure 5:**
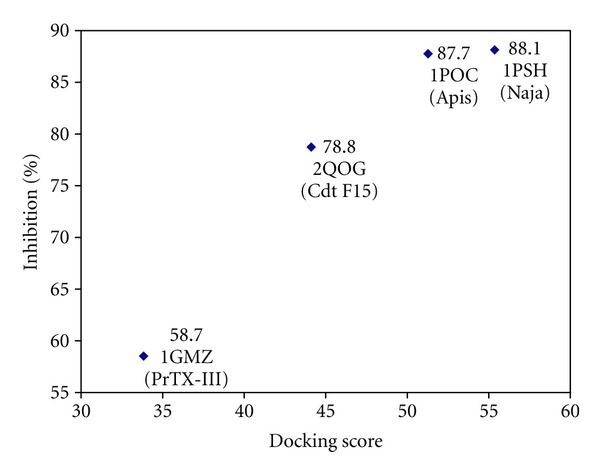
Trend between docking scores performed by GOLD 4.0 (*in silico*) and percentages of inhibition of the enzymatic activity (*in vitro*). The values near the points represent the percentage of inhibition for each PLA2, with the respective PDB ID below. The PDB ID: 1GMZ, 2QOG, 1PSH, and 1POC were used, respectively, for PrTX-III, Cdt F15, Naja, and Apis.

**Figure 6:**
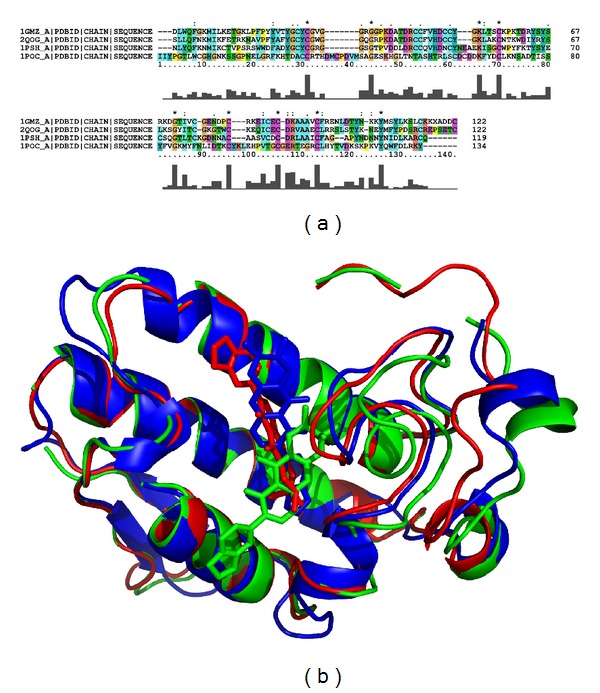
(a) Multiple sequence alignment of PrTX-III, Cdt F15, Naja and Apis, with the PDB ID: 1GMZ, 2QOG, 1PSH and 1POC, respectively. ClustalX was used with default setup. (b) Structure alignment of the PLA2 (cartoon model) with their respective docking solutions for harpalycin 2 (stick model): PrTX-III (green), Cdt F15 (red), and Naja (blue). Figure generated using PyMOL [28].

**Figure 7:**
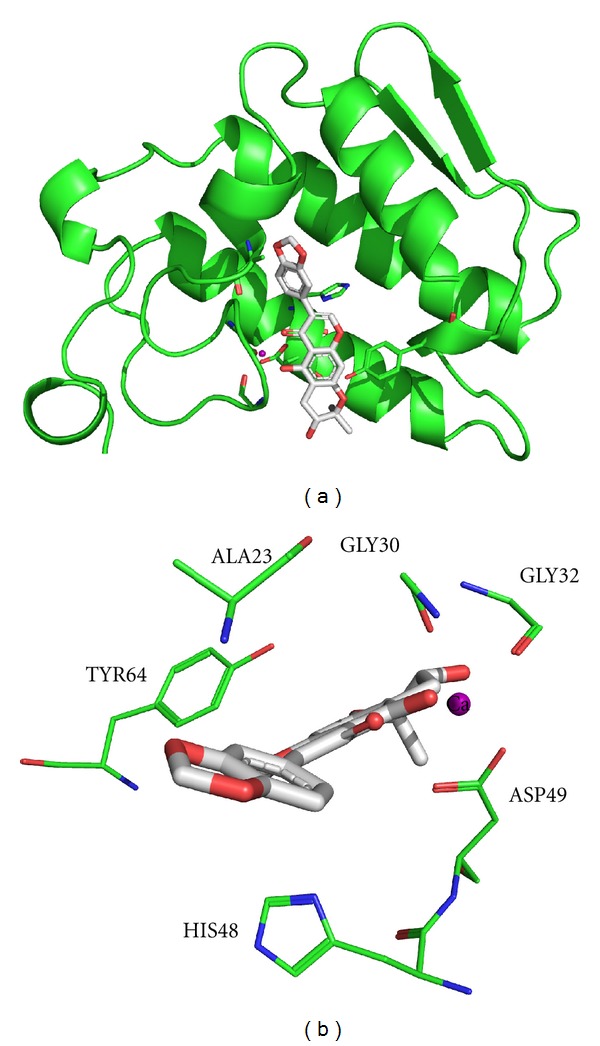
Panoramic (a) and detailed (b) view of the best docking solution obtained with the 1PSH (Naja) target. Important residues, directly involved in intermolecular interactions with the inhibitor (harpalycin 2), are labeled. The Ca^2+^ atom of the catalytic center was also represented in the figure.

The sPLA_2_ structures were represented by the PDB IDs 1GMZ, 2QOG, 1PSH, and 1POC, respectively, for PrTX-III, Cdt F15, Naja, and Apis. The calculated docking score values (GOLD scores) for these targets were 33.93, 44.12, 55.26, and 51.23, respectively, for PrTX-III, Cdt F15, Naja, and Apis. One can see in Figure 5 that an interesting trend between the *in silico* (docking scores) and *in vitro* (percentages of inhibition) results was observed. This means that greater stability (the most positive docking score values) of the complex between the harpalycin 2 and the four sPLA_2_ enzymes is related to greater inhibition percentage of the enzymatic activities. Thus, harpalycin 2 has inhibitory capacity against the enzymatic, edematogenic, and myotoxic activities from neurotoxic venom secretory phospholipases A_2_, probably due to the interaction with residues 48 and 49 in the active site of these toxins. Comparing the amino acid sequences alignment performed by Clustal X [27] among the four sPLA_2_, we found that the Apis has a more specific sequence (with an identity of 24% as compared with PrTX-III) while the other three sequences are similar to each other (identity values of 44% for Naja and 57% for Cdt F15, when compared to PrTX-III), Figure 6(a). Considering the secondary and tertiary structures, the difference between the Apis and the others is more relevant. Therefore, we performed a structural alignment only for PrTX-III, Cdt F15, and Naja, using PyMOL [28], as shown in Figure 6(b). The great similarity among these three PLA_2_ is probably related to their evolutionary origin from snake venoms, where PrTX-III and Cdt F15 are classified as GIIA, while Naja is classified as GIA). Using the 1PSH (Naja) target as example, because it represents the best result, a detailed inspection for the molecular reasons of the good inhibition behavior of the harpalycin 2 in this target can be found in Figure 7. The most important residues in the active site, including HIS48 and ASP49, are labeled and are involved in polar interactions with harpalycin 2.

This data corroborates both ethnopharmacological uses of this plant by Brazilian northeast population: the treatment of snake bites, as sPLA_2_ s are the main toxins in these venoms and, and as anti-inflammatory, due to the main role of sPLA_2_ in the inflammatory cascade of events. The data also pointed to a possible anti-inflammatory activity of this isoflavone mainly in disorders which involve sPLA_2_, such as asthma and rheumatoid arthritis.

## Supplementary Material

The results of CD spectra and chromatographic analysis between harpalycin 2 and the four venom phospholipases A2 tested here are described in Supplementary material.

## Figures and Tables

**Table 1:** Inhibition of enzymatic activity of proinflammatory enzymes by harpalycin-2.

Enzymes	IC_50_ (*μ*g/mL)
PrTX-III	11.34
BPPLA_2_	11.90
HGVPLA_2_	27.42
COX-1	131.90
COX-2	32.73
LOX 15HRC	55.96
LOX 15SY	N/D

Note: PrTX-III (piratoxin-III); BPPLA_2_ (bovine pancreas PLA_2_); HGVPLA_2_ (human group V PLA_2_); COX-1 (cyclooxygenase-1); COX-2 (cyclooxygenase-2); LOX 15HRC (Lipoxygenase 15hrc); LOX 15SY (Lipoxygenase 15sy). N/D (Not determined).

## Abbreviations

Cdt F15: Crotoxin B from *Crotalus durissus* terrificus
COX-1/2: Cyclooxygenase-1/2
CK: Creatine kinase
gVPLA_2_: Group V phospholipase A_2_
Har2: Harpalycin 2
LOX: Lipoxygenase
PAF: Platelet activation factor
PLA_2_: Phospholipase A_2_
PrTX-III: Piratoxin-III
sPLA_2_: Secretory phospholipase A_2_.
