# Supplementary material for: Inhibition of Neurotoxic Secretory Phospholipases A2 Enzymatic, Edematogenic, and Myotoxic Activities by Harpalycin 2, an Isoflavone Isolated from Harpalyce brasiliana Benth
Source: Evid Based Complement Alternat Med. 2012 Jul 31;2012:987517. doi: 10.1155/2012/987517 (PMC3415135; doi:10.1155/2012/987517)

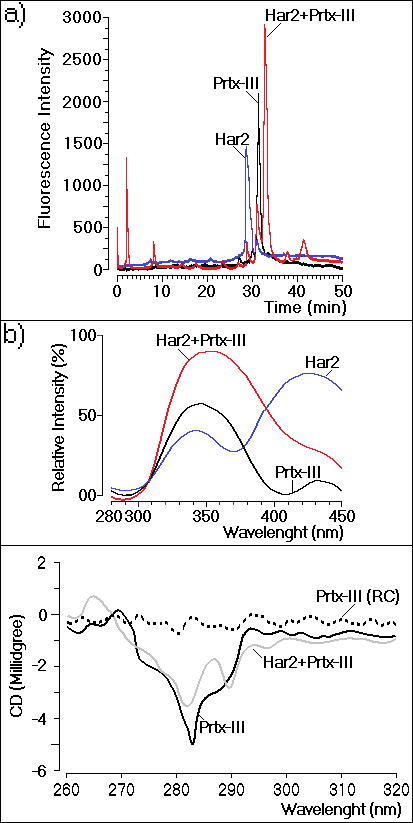
Supp. **1** In figure 1a, chromatographic profile of the Har2, Prtx-III and Har2+Prtx-III obtained from reverse phase HPLC analysis using a C5 column (1x10cm). All runs were done using the same discontinuous gradient of buffer (Acetonitrile 66% in TFA 0.1%) and the chromatographic monitoring was made using a fluorescence detector adjusted for the scanning of tryptophan (Fig. 1b). In Figure 1c, we performed a second time of analysis under the same chromatographic conditions used for the first analysis of Prtx-III and Har2+Prtx-III. Under this condition we performed a CD spectrum in near UV region (320-260 nm) analysis for both proteins.

Supp. **2** In figure 2a, chromatographic profile of the Har2, Cdt F15 and Har2+Cdt F15 obtained from reverse phase HPLC analysis using a C5 column (1x10cm). All runs were done using the same discontinuous gradient of buffer (Acetonitrile 66% in TFA 0.1%) and the chromatographic monitoring was made using a fluorescence detector adjusted for the scanning of tryptophan (Fig. 2b). In Figure 2c, we performed a second time of analysis under the same chromatographic conditions used for the first analysis of Cdt F15 and Har2+Cdt F15. Under this condition we performed a CD spectrum in near UV region (320-260 nm) analysis for both proteins.


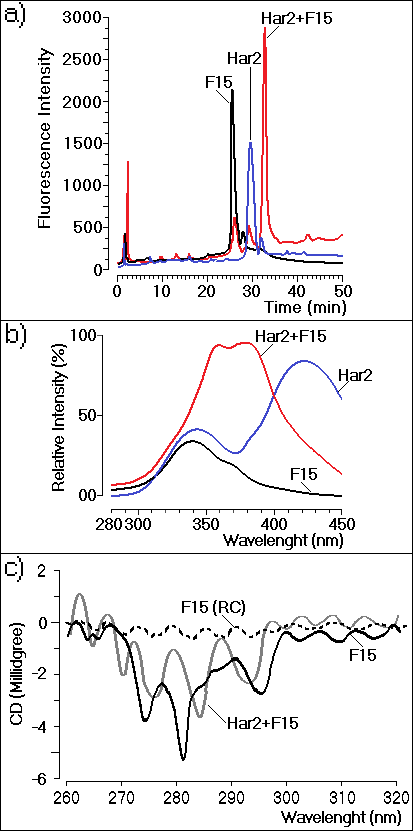


Supp. **3** In figure 3a, chromatographic profile of the Har2, Apis and Har2+Apis obtained from reverse phase HPLC analysis using a C5 column (1x10cm). All runs were done using the same discontinuous gradient of buffer (Acetonitrile 66% in TFA 0.1%) and the chromatographic monitoring was made using a fluorescence detector adjusted for the scanning of tryptophan (Fig. 3b). In Figure 3c, we performed a second time of analysis under the same chromatographic conditions used for the first analysis of Apis and Har2+Apis. Under this condition we performed a CD spectrum in near UV region (320-260 nm) analysis for both proteins.


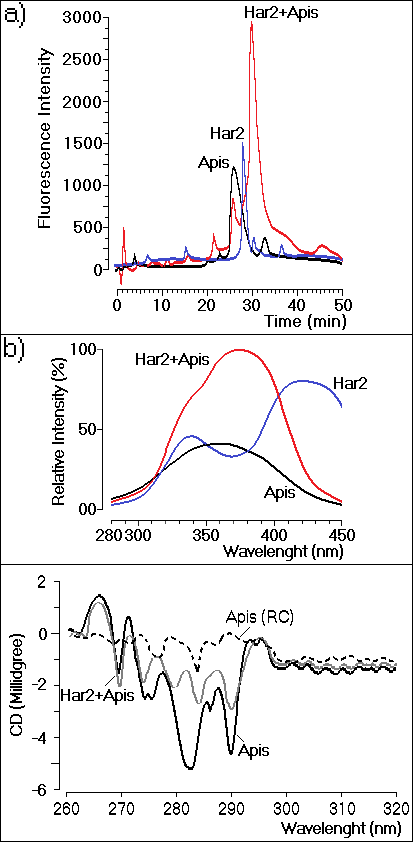


Supp. **4** In figure 4a, chromatographic profile of the Har2, Naja and Har2+Naja obtained from reverse phase HPLC analysis using a C5 column (1x10cm). All runs were done using the same discontinuous gradient of buffer (Acetonitrile 66% in TFA 0.1%) and the chromatographic monitoring was made using a fluorescence detector adjusted for the scanning of tryptophan (Fig. 4b). In Figure 4c, we performed a second time of analysis under the same chromatographic conditions used for the first analysis of Naja and Har2+Naja. Under this condition we performed a CD spectrum in near UV region (320-260 nm) analysis for both proteins.


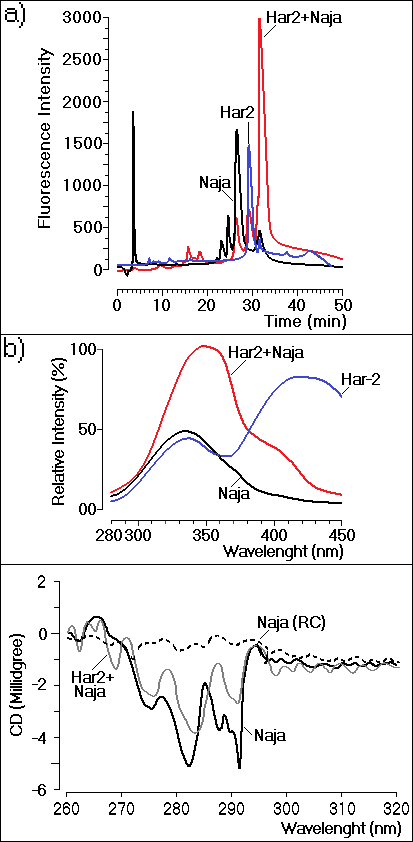

Supplement: Supplementary file 1 — The results of CD spectra and chromatographic analysis between harpalycin 2 and the four venom phospholipases A2 tested here are described in Supplementary material. [file 987517.f1.doc]
